# Level of Vitamin E in Follicular Fluid and Serum and Oocyte Morphology and Embryo Quality in Patients Undergoing IVF Treatment 

**Published:** 2017-06

**Authors:** Mohammad Hadi Bahadori, Seyedeh Hajar Sharami, Fereshteh Fakor, Forozan Milani, Davoud Pourmarzi, Seyedeh Fatemeh Dalil-Heirati

**Affiliations:** 1 Department of Anatomy, Guilan University of Medical Sciences, Rasht, Iran; 2 Reproductive Health Research Center, Guilan University of Medical Sciences, Rasht, Iran

**Keywords:** Vitamin E, Follicular Fluid, IVF, Oocyte Morphology, Embryo Quality

## Abstract

**Objective:** This study aimed to determine the relationship between vitamin E in the follicular fluid (FF) and serum with oocyte morphology and embryo quality.

**Materials and methods:** A cross-sectional study was conducted on serum samples, FF samples, oocytes, and embryos collected from 50 women undergoing *in vitro* fertilization in the Alzahra Hospital, Rasht, Iran from March to August 2014. Vitamin E level was measured using HPLC. Oocyte morphology and embryo quality were evaluated during inverted optical microscopy.

**Results:** Totally 434 oocytes and 199 embryos were examined. Most frequently the metaphase II (MII) oocytes were observed at the 0.35-1 mg/dl level of vitamin E in FF (89.2%) and the 1-5 mg/dl level of vitamin E in serum (86.1%). The odds of having MII oocytes at the level of 0.35-1 mg/dl (OR = 2.48, 95% CI = 1.24-4.94) and 1.5-2 mg/dl (OR = 2.51, 95% CI = 1.02-6.19) of vitamin E in FF was significantly higher compared to level of 2-7.4 mg/dl. The effect of vitamin E serum level on oocyte maturation was not significant. The odds of having embryo with Z1 or Z2 quality, at the 10-15 mg/dl level of vitamin E in serum (OR = 6.45, 95% CI = 1.18-35.22), compared to the 15-20 mg/dl level, was significantly higher. The effect of vitamin E levels in FF on the embryo quality was not significant.

**Conclusion:** At certain levels of vitamin E in the FF, oocytes with higher maturation and at certain levels of vitamin E in serum, embryo with higher quality can be achieved.

## Introduction

Although, various recent investigations assessed the use of assisted reproductive technology (ART) and led to its evolution (1). But with all the advances still a considerable percentage of infertile couples who are treated with high costs and use of ART procedures do not experience a successful pregnancy (2). Worldwide efforts to achieve the highest success rate in ART are underway (3). *In vitro* fertilization (IVF) is one of the most common ART. Many factors can effect on the process of IVF and then in the success of infertility treatment with this method (4). Sperm and oocyte quality before fertilization and embryo quality before the transfer to the uterus are of great importance in IVF success (4-6). Follicular fluid (FF) is an important environment for the development of oocytes. Increase or decrease in compositions of the liquid impacts on oocyte morphology and embryo quality (7, 8). Adverse effects associated with oxidative stress and reactive oxygen species (ROS) in the body and *in vitro* on the quality of sex cells is approved. Reactive oxygen species in culture medium of embryos in IVF reduces embryo quality and increases the amount of fragmentation and thereby can reduce the chances of reproduction success (9). Vitamins can affect the reproductive system of men and women through an oxidative mechanism and activity of antioxidants that reduce the excessive production of free radicals in infertile men and women (10, 11). Little research about the effect of antioxidants on humans’ fertility are available. However, few studies have been done on the effects of some dietary supplements and multivitamins and antioxidants on growth and quality of oocytes and embryos (9, 12). Vitamin E is among the most important natural antioxidants that protect cells from damage caused by free radicals. Vitamin E deficiency causes sterility in male animals and decreased fertility or the failure to end the pregnancy in mice (12, 13). Therefore, an adequate level of vitamin E is considered necessary for reproduction in animals (8). According to the results of studies on animals and antioxidant effects of vitamin E, it seems that the development of oocyte and human embryo is affected by the concentration of this vitamin in FF and serum (10, 13, 14). Despite numerous studies conducted on animals there are too little knowledge about the effects of antioxidants such as vitamin E on the morphology of oocyte and embryo quality in human. This study aimed to determine the relationship between vitamin E concentration in the FF and serum with morphology of oocyte, and embryo quality in women undergoing IVF treatment.

## Materials and methods

***Setting and subjects: ***This cross-sectional study was conducted on 50 women undergoing IVF in infertility clinic of Alzahra Hospital, Rasht, Iran during March 2014- August 2014. This study was approved by the ethics committee of Guilan University of Medical Sciences with number code 1930162908 and all patients provided written informed consent before entering the study. Inclusion criteria were age between 20 and 45 years and female smokers were excluded. Data including men and women’s age, duration of infertility, cause of infertility, and history of taking supplements containing vitamin E in the past two months were collected by asking patients. Weight and height were measured and women’s BMI was calculated.

***Ovulation induction: ***All of the patients were undergone similar ovulation induction protocol (gonadotropin-releasing hormone agonist or antagonist). Using transvaginalsonography follicle growth was measured every other day, and if necessary, their serum levels of estradiol were measured as well. Ten thousand units of human chorionic gonadotropin (HCG) were injected to all patients 36 hours before the oocyte retrieval.

***Extraction of follicles and FF: ***Given the size of the follicles, oocyte retrieval was performed on days 10 to 14 of the menstrual cycle. Oocytes were collected after 32 to 36 hours after the HCG injection. In follicle extraction through vaginal sonography, the ovaries were first examined and then suctioning was done.

During puncture procedure to remove the oocyte from the ovary, the follicle of at least 18 mm in diameter together with FF were aspirated by catheter and the this collection were collected into the test tube and then pored into the plates. The oocytes after being washed in GMOPSPLUS (Vitrolife, Sweden), were held for 2 to 4 hours in incubator at temperature of 37° C and CO2 (as much as 6%).

Then naked oocytes were kept in dishes containing the G1 V5^PLUS^ (Vitrolife, Sweden) for IVF inside the incubator. Follicular fluid by falcon tubes at 20° C after the suction were maintained and transferred to the laboratory in order to avoid exposure to light, the tubes were enclosed in aluminum foil.

***Assessing oocyte morphology, fertilization and embryo quality: ***Using an inverted microscope, oocytes were categorized in three groups of metaphase II (MII) oocytes, metaphase I (MI) oocytes, germinal vesicle (GV) oocytes, and degenerated oocyte. Fertilization assessment was done 15 to 20 hours after adding sperm. Male and female pronucleuses formation was a means for verification of fertilization. For embryo transfer, only 2-pronuclei (2PN) were used. The zygotes were scored using the Z-score scoring system. In this system Z1zygotes have equal numbers of arranged nucleoli in contrast of the adjacent pronuclei. Z2 zygotes have equal numbers of nucleoli with the similar size arranged in a nucleus on the side section but scattered in the other nucleas. Z3 zygotes have equal sizes and numbers of nucleoli in two nuclei, with one nucleus having aligned nucleoli at the pronuclear junction and the other having scattered nucleoli in itself. Zygotes with unequal numbers and/or sizes of nucleoli are also considered as *Z*3. Z4 zygotes that are unarranged pronuclei and have slightly different sizes have not been located at the center of the zygote (15, 16).

***Measurement of vitamin E*** *: *Five ml blood sample before extracting the oocyte and 1 ml FF was taken from all subjects. Level of vitamin E was measured using High Performance Liquid Chromatography (HPLC) (IBL, Germany). All measurements were done in a predetermined laboratory.

***Statistical analysis: ***To make comparisons between different groups of oocyte morphology and embryo quality, the obtained oocytes were divided to two groups of MII oocytes (mature oocytes) and other oocytes (GV + MI + degeneration) oocytes. The resulting embryos in the first day were divided to two groups with Z1 or Z2 and Z3 or Z4 quality groups. To investigate the effect of infertility on oocyte morphology and embryo quality, infertile women (due to female and combined infertility) with healthy women (causes of male infertility, unknown, and donator) and infertile men (causes of male infertility or combination infertility) with healthy men (causes of female infertility and unknown) were compared.

The data were analyzed by SPSS software version 21.0 (SPSS, SPSS Inc., Chicago, IL, USA). Continuous data were shown as Mean ± SD, and categorized data as number (percentage). To compare the frequency between the groups, the chi-square test and Fisher's exact test were used. For comparing the mean between the two groups the independent *t* test was used. Pearson correlation coefficient was used to test the correlation between the level of vitamin E with the number of oocytes and embryos and between the levels of vitamin E in FF and serum. To evaluate the net effect of variables under study on oocyte morphology and embryo quality, logistic regression (Forward: LR) was used. A P value less than 0.05 was considered statistically significant.

## Results

***Feature of obtained oocytes: ***From 50 under study women, after ovarian stimulation 434 oocytes were obtained. The average number of oocytes was 8.66 ± 5.23 with range of 1 to 24. The highest numbers of oocytes, i.e., 349 cases (80.4%) were MII, 49 cases (11.3%) were MI, 23 cases (5.3%) were GV, and 13 cases (3%) were degenerated.

***Comparing studied variables between the two groups of oocytes morphology: ***The frequency of MII oocytes was significantly higher in women with higher age (p = 0.006), infertile women (p = 0.0001), and women who were taking supplementation contain vitamin E (p = 0.010). Differences in frequency MII oocytes among different groups of women’s BMI and duration of infertility were not significant (Table 1).

***The level of vitamin E in FF and its relationship with oocyte morphology: ***The correlation between the level of vitamin E in FF with the total number of oocytes (r = 0.159, p = 0.269) was not statistically significant. 

The difference of means level of vitamin E in FF between the two groups of MII oocytes (2.39 ± 1.97 mg/dl) and other oocytes (2.48 ± 1.52 mg/dl) was not statistically significant (p = 0.709). The frequency of MII oocytes and MI, GV and degenerate oocytes in different levels of vitamin E was compared with Chi-square test.

**Table 1:** Comparing frequency of MII oocytes and other oocytes at different groups of studied variables

**Variables**		**MII**	**MI, GV, Degenerated**	**p value**
Women age	23-29	128 (73.6%)	46 (26.4%)	0.006
	30-34	116 (82.3%)	25 (17.7%)	
	35-44	105 (88.2%)	14 (11.8%)	
Women BMI	18-24.9	103 (82.4%)	22 (17.6%)	0.771
	25-29.9	157 (80.1%)	39 (19.9%)	
	30≤	89 (78.8%)	24 (21.2%)	
Infertility duration (year)	5˃	108 (85.7%)	18 (14.3%)	0.084
	5≤	241 (78.2%)	67 (21.8%)	
Female factor infertility	Yes	152 (90.5%)	16 (9.5%)	0.0001
	No	197 (74.1%)	69 (25.9%)	
Use of supplement contain vitamin E	Yes	125 (87.4%)	18 (12.6%)	0.010
	No	224 (77%)	67 (23%)	

**Table 2:** Comparing the frequency distribution of MII oocytes and other oocytes between different levels of vitamin E in FF and serum

**Vitamin E level** mg/dl		**MII N (%)**	**MI, GV, Degenerated N (%)**	**p value**
Follicular fluid	0.35-1	124 (89.2)	15 (10.8)	0.002
	1-1.5	55 (69.6)	24 (30.4)	
	1.5-2	45 (84.9)	8 (15.1)	
	2-7.4	125 (76.7)	38 (23.3)	
Serum	1-5	62 (86.1)	10 (13.9)	0.297
	5-10	214 (79)	57 (21)	
	10-15	49 (84.5)	9 (15.5)	
	15-20	24 (72.7)	9 (27.3)	

Given that the minimum level of vitamin E in our study samples was 0.35 mg/dl, division of classes began from 0.35. The highest observed level of vitamin E was 7.4 mg/dl and considered as the upper limit of the last class. The highest percentage of MII oocytes (89.2%) was observed in the 0.35-1 mg/dl level of vitamin E. The lowest percentage of MII oocyte was observed in the 1-1.5 mg/dl level of vitamin E. Difference in frequency distribution of oocytes maturity among different groups of vitamin E level was significant (p = 0.002) (Table 2).

***Vitamin E serum level and its relationship with oocyte morphology: ***The correlation between serum vitamin E level with the total number of oocytes (r = 0. 104, p = 0. 472) was not statistically significant. The difference of means of serum vitamin E level between the two groups of MII oocytes (7.61 ± 3.89 mg/dl) and other oocytes (7.89 ± 4.09 mg/dl) was not significant (p = 0.554).

The frequency of MII oocytes and MI, GV and degenerated oocytes in different levels of serum vitamin E were compared using the chi-square test. Given that the minimum level of vitamin E in this study samples was 1 mg/dl, the division of classes began from 1. The highest level of observed vitamin E was 20 mg/dl, which was considered as the upper limit of the last class. The highest percentage of MII oocyte (86.1%) was observed in 1-5 mg/dl level of vitamin E. The lowest percentage of MII oocyte was observed in the level of vitamin E 20-51 mg/dl. But this difference in frequency distribution was not statistically significant (p = 0.297) (Table 2).

***The net effect of vitamin E on the oocyte morphology: ***Based on the results of the regression analysis, the odds of having MII oocytes in women aged 23-29 years compared with age group 35-44 years was significantly lower (p = 0.0001). But this reduction of chance for women aged 30-34 years compared with age group 35-44 years was not significant (p = 0.088). The odds of having MII oocytes in fertile women (p = 0.0001), and women without history of taking supplement contain vitamin E (p = 0.018) were significantly lower. The odds of having MII oocytes at the level of 0.35-1 mg/dl (OR = 2.48, 95% CI = 1.24-4.94) and 1.5-2 mg/dl (OR = 2.51, 95% CI = 1.02-6.19) of vitamin E in FF compared to level of 2-7.4 mg/dl was significantly higher (Table 3).

**Table 3:** Regression analysis of factors affecting oocyte morphology

**Variables**		**OR**	**95% confidence intervals**	**p value**
Women age	35-44	Reference		
	30-34	0.51	0.24-1.11	0.088
	23-29	0.27	0.13-0.56	0.0001
Female infertility	Yes	Reference		
	No	0.31	0.17-0.59	0.0001
Use of supplement contain E vitamin	Yes	Reference		
	No	0.45	0.23-0.88	0.018
E vitamin levels	2-7.4	Reference		
	1.5-2	2.51	1.02-6.19	0.045
	1-1.5	1.35	0.66-2.75	0.407
	0.35-1	2.48	1.24-4.94	0.010

**Table 4:** Comparing the frequency of embryo with Z1 or Z2 quality and Z3 or Z4 quality between different groups of studied variables

**Variables**		**Z1 , Z2**	**Z3 , Z4**	**p value**
Women age	23-29	46 (56.1%)	36 (43.9%)	0.555
	30-34	39 (60.9%)	25 (39.1%)	
	35-44	27 (50.9%)	26 (49.1%)	
Men age	35 ≥	57 (57%)	43 (43%)	0.887
	35 ˂	55 (55.6%)	44 (44.4%)	
Women BMI	18-24.9	37 (53.6%)	32 (46.4%)	0.821
	25-29.9	51 (58.6%)	36 (41.4%)	
	30 ≤	24 (55.8%)	19 (44.2%)	
Infertility duration (year)	5 ˃	52 (66.7%)	26 (33.3%)	0.020
	5 ≤	60 (49.6%)	61 (50.4%)	
Female infertility	Yes	47 (58.8%)	33 (41.3%)	0.662
	No	65 (54.6%)	54 (45.4%)	
Male infertility	Yes	58 (70.7%)	24 (29.3%)	0.001
	No	54 (46.2%)	63 (53.8%)	
Use of supplement contain vitamin E	No	72 (55.8%)	57 (44.2%)	0.882

***Feature of developed embryos: ***One hundred and ninety nine embryos were obtained from 45 women. From 5 cases, no embryo was obtained. The mean number of embryos was 3.98 ± 2.98 with range of 1 to 13. Majority of embryo (102 cases, 51.3%) had Z2 quality, 10 cases (5%) had Z1 quality, 75 cases (37.7%) had Z3 quality, and 12 cases (6%) had Z4 quality.

***Comparing studied variables between the two groups of embryo quality: ***Those with infertility duration less than 5 years had significantly higher quality embryos (p = 0.020). In infertile men greater percentage of obtained embryos were with Z1 or Z2 qualities significantly (p = 0.001). The differences of frequency distribution of embryos with Z1 or Z2 quality among the different female age, male age, women’s BMI, women’s infertility, and taking supplement contain vitamin E groups were not statistically significant (Table 4).

***Vitamin E level in FF and its relation to embryo quality: ***The correlation between vitamin E level in FF with the total number of 2PN embryos (r = 0.028, p = 0.848) was not significant. The difference of means level of vitamin E between the two groups with Z1 or Z2 quality (2.35 ± 0.2.12 mg/dl) and Z3 or Z4 quality (2.21 ± 1.87 mg/dl) was not statistically significant (p = 0.641). The most frequency of embryo with Z1 and Z2 quality (64%) was observed in the 1.5-2 mg/dl level of vitamin E. Difference in frequency distribution of embryo quality among different levels of vitamin E in FF was not significant (p = 0.117).

***Serum vitamin E Level and its relation to embryo quality: ***The correlation between serum vitamin E level with the total number of 2PN embryos (r = -0.008, p = 0.958) was not significant. The difference of means level of vitamin E between the two groups with Z1 or Z2 quality (7.68 ± 3.45 mg/dl) and Z3 or Z4 quality (7.14 ± 4.14 mg/dl) was not significant (p = 0.318). The most frequency of embryo with Z1 and Z2 quality (87.5%) was observed in the 10-15 mg/dl level of vitamin E. Difference in frequency distribution of embryo quality among different levels of vitamin E serum was statistically significant (p = 0.007) (Table 5).

**Table 5:** Comparing embryo frequency with Z1 or Z2 quality and Z3 or Z4 quality in different groups of vitamin E level in the FF and serum

**Vitamin E level mg/dl l**		**Z1, Z2 N (%)**	**Z3, Z4 N (%)**	**p value**
Follicular	0.35-1	52 (61.9)	32 (38.1)	0.117
	1-1.5	10 (37)	17 (63)	
	1.5-2	16 (64)	9 (36)	
	2-7.4	34 (54)	29 (46)	
Serum	1-5	18 (46.2)	21 (53.8)	0.007
	5-10	67 (54.9)	55 (45.1)	
	10-15	21 (87.5)	3 (12.5)	
	15-20	6 (42.9)	8 (57.1)	

**Table 6:** Regression analysis of the factors affecting embryo quality

**Variables**		**OR**	**95% confidence intervals**	**p value**
Infertility duration	5≤	Reference		
	5˃	2.34	1.20-4.56	0.012
Male infertility	Yes	Reference		
	No	0.297	0.15-0.57	0.0001
Serum level of vitamin E	15-20	Reference		
	10-15	6.45	1.18-35.22	0.031
	5-10	1.48	0.45-4.90	0.517
	1-5	0.82	0.22-3.09	0.764

***The net effect of vitamin E on embryo quality: ***Based on the results of logistic regression analysis, the odds of having an embryo with Z1 or Z2 quality in couples with infertility duration less than 5 years was significantly higher than for couples with infertility duration higher than 5 years (p = 0.012). Odds of having an embryo with Z1 or Z2 quality in infertile male was significantly higher than fertile male (p = 0.0001). Odds of having embryo with Z1 or Z2 quality at the 10-15 mg/dl serum level of vitamin E (OR = 6.45, 95% CI = 1.18-35.22) compared with the 15-20 mg/dl level was significantly higher. In other levels of vitamin E, differences were not significant (Table 6).

***The correlation between vitamin E level in the FF and serum: ***Significant direct correlation was found between level of vitamin E in the FF and serum (r = 0.380, p = 0.006).

## Discussion

The effect of diet on the development of oocytes and embryo is approved, the lack of many vitamins and nutrients can reduce the chances of successful natural fertility (17). Follicular fluid is an important factor in the development of oocyte and the concentrations of some compounds of FF are related to oocyte and embryo quality (7, 8). In this study, the most percentage of MII oocytes was observed at 0.35-1 mg/dl level and then at 1.5-2 mg/dl level of vitamin E in FF. In regression analysis, the odds of having MII oocytes in these two levels of vitamin E compared with the 2-7.4 mg/dl level of vitamin E was significantly higher and about 2.5 times. But significant relationship between serum vitamin E level and the oocyte maturation was not found. Also in this study, there was no relationship between vitamin E levels in FF with embryo quality, but in the 10-15 mg/dl level of vitamin E serum, the most frequent embryo with Z1 and Z2 quality (87.5%) had been observed. Besides, the odds of having Z1 or Z2 embryo in 15-10 mg/dl serum level of vitamin E compared to 15-20 mg/dl level was about 6.5 times. 

In a study by Wang et al. that investigated the effect of vitamin E in reducing oxidative stress toxicity of mouse embryo blastocyst development, it was concluded that to avoid the adverse effects of ROS in mouse embryo, the protective effect of antioxidant-containing supplements (e.g., Vitamin E and C) is needed (14). According to Micheal et al.’s study, conducted to examine the effect of alphatocopherol and ascorbic acid combination in improving sheep oocyte maturation and cumulus expansion of oocytes, it was shown that the combination of 20 mM of alphatocopherol and 750 mM of ascorbic acid has beneficial effects on cytoplasmic maturation (18). Thigagarajan et al. investigated the effect of vitamin E on the development of Buffalo embryo *in vitro*. In that study, concentrations 0, 50, 100, 200 and 400 mM of vitamin E in culture medium were examined and shown that these concentrations of vitamin E, did not significantly affect the oocyte maturation. But in the first 72 hours, the blastocyst rate and the total number of cells in 100 mM group was significantly increased compared with the control group (19). In Biswasetal.s’ study, the effects of increasing vitamin E in the diet of Indian Native Kadaknath Hen (NKH) were evaluated on the egg production and quality traits. In chickens aged 180 days for 30 weeks in groups of 20 chicks, distribution of vitamin E has conducted as (15, 150, 300 IU/kg).  It was shown that the dietary supplement vitamin E with an average level of 150 IU/kg resulted in production performance and productivity in Indian NKH and improving the quality traits of egg including albumen and yolk weight and shell weight. But body weight and egg weight and breeding in these conditions did not change significantly (20). In Olson et al.’s study in investigating the effect of vitamin E in the development of cattle embryo in vitro, it was shown that cattle embryo blastocyst development rate is increased up to 63% at morula stage with an increase as 100 mM in Vitamin E (21). In a study by Shariatzadeh et al. conducted to examine the effect of vitamin E on ocyte construction in rats treated with sodium arsenite, it was shown that in rats taken sodium arsenite and vitamin E, compared to those who only taken sodium arsenite, the average size of the corpus luteum, oocyte size in a variety of primordial, primary and secondary follicles, and the mean volume of oocyte nuclear in different types of primer, secondary and antral and graph follicles a significant increase was shown (22).

***Conclusion: ***According to findings of this study, at 0.35-1mg/dl and 1.5-2 mg/dl levels of vitamin E in FF, the highest percentage of MII oocytes can be achieved, but there is no relationship between the level of vitamin E serum and oocyte maturation. But in 10-15 mg/dl serum level vitamin E, the highest percentage of higher quality of embryos can be obtained. It seems that at a certain levels of vitamin E in the FF, we can have higher maturated oocytes. Also, in a certain level of vitamin E serum, we can achieve high-quality embryos.

It is recommended that, further studies to be done with investigating the effect of other antioxidants on oocyte maturation and embryo quality as well as examining the relationship between vitamin E levels in FF and serum with a success rate of fertility. Also results of this study can be useful to choose proper dose in interventional studies.
